# An effective biocompatible fluorescent probe for bisulfite detection in aqueous solution, living cells, and mice†

**DOI:** 10.1039/d0ra03329d

**Published:** 2020-07-03

**Authors:** Ruqiao Zhou, Guiling Cui, Yuefu Hu, Qingrong Qi, Wencai Huang, Li Yang

**Affiliations:** State Key Laboratory of Biotherapy and Cancer Center, West China Hospital, Sichuan University Chengdu Sichuan 610041 P. R. China yangli@scu.edu.cn +86-159-2818-5249 +86-159-2818-5249; West China School of Pharmacy, Sichuan University Chengdu Sichuan 610041 China; School of Chemical Engineering, Sichuan University Chengdu Sichuan 610065 China

## Abstract

Sulfur dioxide, an air pollutant, is easily hydrated to sulfites and bisulfites and extremely harmful to human health. On the other hand, endogenous sulfur dioxide is the fourth gasotransmitter. In view of the above, it is worth developing an effective method for the detection of these compounds. In this paper, a novel colorimetric fluorescent probe (Hcy-Mo), based on hemi-cyanine, for bisulfites is reported. Hcy-Mo shows excellent selectivity for bisulfites over various other species including cysteine, glutathione, CN^−^, and HS^−^, and undergoes 1,4-addition reactions at the C-4 atom of the ethylene group. The reaction can be completed in 30 s in a PBS buffer solution and displays high sensitivity (limit of detection is 80 nM) for bisulfites. Test paper experiments show that the probe can be used for bisulfite detection in aqueous solutions. In addition, Hcy-Mo exhibits excellent cell permeability and low cytotoxicity for the successful detection of bisulfites in living MDA-MB-231 cells and in living mice, implying that this probe would be of great benefit to biological researchers for investigating the detailed biological and pharmacological functions of bisulfites in biological systems.

## Abstract 2

Sulfur dioxide, an air pollutant, is easily hydrated to sulfites and bisulfites and extremely harmful to human health.

## Graphical abstract

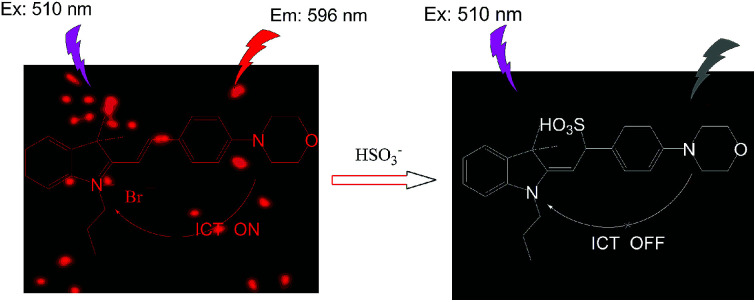

## Introduction

Bisulfites are widely used in the food, beverage, and pharmaceutical industries as essential preservatives due to their anti-oxidation properties and ability to inhibit microbial growth and prevent discoloration.^1,2^ However, pathological studies have found that high concentrations of bisulfites are associated with inflammation,^3–6^ respiratory diseases,^7^ chromosomal aberrations,^8,9^ system diseases,^10–13^ and even cancer.^14^ On the other hand, sulfur dioxide, one of the most well-known air pollutants,^15,16^ can dissolve in water and form an equilibrium state between the sulfites and bisulfites. However, recently researchers have found that endogenous sulfur dioxide, which is present as bisulfites *in vivo*, is the fourth gasotransmitter similar to carbon monoxide (CO), nitrogen monoxide (NO), and hydrogen sulfide (H_2_S).^17,18^ Thus, in view of the effects of the sulfites mentioned above and the significance of bisulfites *in vivo*, the intake of bisulfites by the human body must be limited. Therefore, it is vital to find a fast and efficient method for *in vivo* bisulfite detection.

There are some conventional detection methods for bisulfites, such as colorimetry, titrimetry, chromatography, and electrochemical analysis.^19–24^ Unfortunately, it is difficult to apply these methods to endogenous bisulfite detection in real time due to the limitations of the methods and equipment. Recently, carbon dots,^25,26^ quantum dots,^27,28^ and fluorescence-based probes have emerged as fluorescent materials for biosensing. Among them, fluorescence-based probes are a promising class due to their non-invasiveness, high sensitivity, and high temporal and spatial resolution.^29–33^ At present, bisulfite fluorescent probes mainly include dinitrile alkenyl,^34,35^ dinitrile alkenyl analogs,^36–38^ aldehyde derivatives,^39–41^ and nitro-containing compounds.^42^ However, these types of probes have some disadvantages, including a long response time, unsatisfactory limits of detection, and interference from biothiols, which limit their application. In contrast, hemi-cyanine has many advantages such as simple preparation, low cost, membrane permeability, organelle targeting and so on.^43–45^ Therefore, it is highly desirable to develop a novel fluorescent probe for biological samples. Many fluorescent probes have previously been reported in the literature,^43,44,46–50^ however, improvements are required for some properties. Therefore, an ideal fluorescent probe for *in vivo* bisulfite detection is still needed.

Herein, a fluorescent Hcy-Mo probe with naked eye recognition of bisulfites was designed and synthesized. Based on a biologically active hemi-cyanine and featuring a morpholine group, Hcy-Mo possessed good cell membrane permeability. The response mechanism can be verified by nuclear magnetic resonance spectroscopy (NMR), that is, the change in molecular conjugation due to the 1,4-addition reaction of the probe and bisulfites. Moreover, Hcy-Mo can be used to rapidly and specifically detect bisulfites in breast cancer cells and living mice.

## Experimental

### Materials and instruments

All of the chemical reagents and solvents were obtained commercially (Adamas Reagents or Cologne Reagents) and used as received without further purification unless otherwise stated. Ultrapure water was purified using a Millipore water purification system. Silica gel P60 (Qingdao, mesh number 200–300) was used for column chromatography. ^1^H NMR and ^13^C NMR spectra were recorded on a Bruker 400 M instrument and chemical shifts are given in ppm using the peak of residual proton signals of DMSO-*d*_6_ or CDCl_3_-*d*_6_ as the internal standard. The mass spectra (ESI) were recorded on a Finnigan LCQ^DECA^ spectrometer with ESI mode. UV-Vis and fluorescence spectra were recorded on a SHIMADZU UV-2450 spectrophotometer and a VARIAN Cary Eclipse FL1003 M013 spectrometer, respectively. The fluorescence images of the MDA-MB-231 cells were recorded on an inverted fluorescence microscope (Olympus, Japan). The fluorescence images of the living mice were recorded on a Night OWL LB983 living imager.

All of the animal procedures were performed in accordance with the Guidelines for Care and Use of Laboratory Animals of Sichuan University and approved by the Animal Ethics Committee of Sichuan University.

### Synthesis of the probe Hcy-Mo

**Scheme 1:**
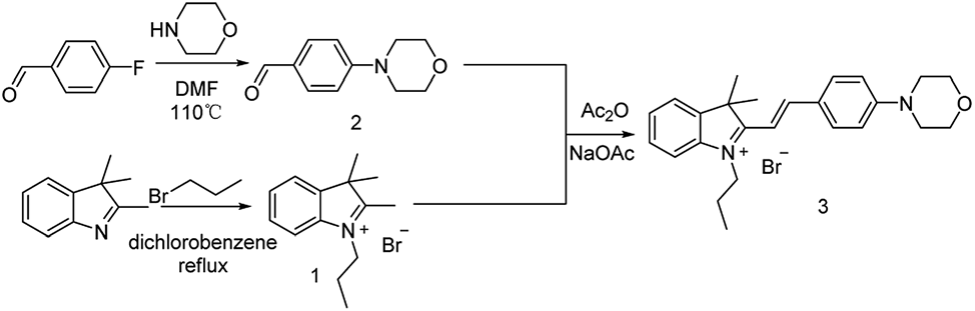
Synthetic route to probe Hcy-Mo.

As shown in Scheme 1, Hcy-Mo was prepared over three steps using 2,3,3-trimethylindolenine as the starting material. Structural identification of the products was confirmed by NMR and HRMS spectrometry.

A mixture of 4-morpholinebenzaldehyde (306 mg, 1.6 mmol), 2,2,3-trimethylsulfonium bromide (421 mg, 1.5 mmol), and anhydrous sodium acetate (120 mg, 1.5 mmol) in 6 mL of anhydrous acetic anhydride was refluxed at 85 °C for 1–2 h under a nitrogen atmosphere. The reaction was monitored by TLC. After completion, the mixture was cooled to room temperature and poured into a saturated brine solution. The product was extracted with dichloromethane 3 times. The combined organic phases were concentrated *in vacuo*. Then, 30 mL of isopropyl ether was added to the solution and the solution was filtered. The filter cake was washed 3–4 times with isopropyl ether. After that, the product was dried *in vacuo* to give a purple solid product 320 mg, yield 46.9%. ^1^H NMR (400 MHz, DMSO-*d*_6_) *δ* 8.37 (d, *J* = 15.8 Hz, 1H), 8.13 (d, *J* = 8.7 Hz, 2H), 7.81 (t, *J* = 7.8 Hz, 2H), 7.57 (t, *J* = 7.2 Hz, 1H), 7.52 (t, *J* = 7.3 Hz, 1H), 7.39 (d, *J* = 15.8 Hz, 1H), 7.11 (d, *J* = 8.7 Hz, 2H), 4.55 (t, *J* = 7.2 Hz, 2H), 3.75 (t, *J* = 4.7 Hz, 4H), 3.51 (t, *J* = 4.8 Hz, 4H), 1.85 (q, *J* = 7.3 Hz, 2H), 1.77 (s, 6H), 1.00 ppm (q, *J* = 7.5 Hz, 3H). ^13^C NMR (100 MHz, DMSO-*d*_6_) *δ* 180.85, 155.10, 154.88, 143.51, 141.51, 134.45, 129.35, 128.51, 124.43, 123.37, 114.65, 113.89, 106.94, 66.27, 51.78, 47.10, 46.87, 26.89, 21.92, 11.27 ppm. ESI-MS *m*/*z* calcd for chemical formula: C_25_H_31_N_2_O [M]^+^: 375.2191; found: 375.2184.

### Spectral experiment

0.91 mg of the Hcy-Mo probe was dissolved in 2 mL of PBS solution to prepare a 1 mM stock solution. The stock solution was then diluted to obtain a test concentration (10 μM). 2.08 mg of sodium bisulfite was dissolved in 2 mL of distilled water to prepare a 10 mM stock solution, which was diluted to prepare the required test concentrations. Interfering ions were selected from different sodium salts (NaF, NaCl, NaBr, NaI, NaHCO_3_, Na_2_CO_3_, CH_3_COONa, Na_2_S_2_O_3_, Na_2_SO_4_, NaNO_2_, NaN_3_, NaSCN, cysteine, glutathione, tetrabutyl cyanamide, and NaHS) for the preparation of 20 mM stock solutions. The stock solutions were diluted to the desired test concentration.

### Cytotoxicity and cell imaging

A 96-well culture plate was inoculated with logarithmic growth phase cells. The cell suspension concentration in the culture dish was adjusted and the edge plate was filled with sterile PBS buffer and placed in an incubator (5% CO_2_, temperature 37 °C). The culture was continued until the cell monolayer was spread over the bottom of the plate. The cells were divided into experimental groups and a control group. Hcy-Mo was not added to the control group. Different gradients of the probe Hcy-Mo solution were added to the experimental groups. After completion, the culture was placed in the incubator (5% CO_2_, temperature 37 °C). After 2 h, 10 μL of the MTT solution was added to each well and the cells were incubated for 24 hours. The absorbance of each well was measured using a microplate reader and the cell survival rate was calculated.

Then, the medium in the well was replaced with a medium containing the probe (probe concentration: 10 μM). After incubation for 1 h, the medium containing the probe solution was discarded and the cells were washed with sterile PBS 2–3 times to remove the non-incorporated cells. After that, the sodium bisulfite solution was added to each well and incubation was continued for 30 min. Then, the sample was inverted under a microscope to observe and collect imaging data using the red fluorescent channel.

### Mice imaging

The Hcy-Mo probe (50 μM) and the test substance sodium bisulfite (500 μM) were prepared in PBS. The 24 hour fasting Kunming mice were divided into a blank group, control group and experimental group. The blank group was injected with PBS, the control group was injected with the probe, and the experimental group was injected with the probe and the test substance. Tail vein injection was adopted as the injection method in this study.

## Results and discussion

### Design of Hcy-Mo

A fluorophore is needed for the design of fluorescent probes. Hemi-cyanine has attracted attention for its mitochondrial targeting.^51–55^ In addition, morpholine is a good electron donor and exhibits lysosome-targeting.^56,57^ Thus, a mitochondrial targeting group is linked to a lysosome-targeting group by a “π” bridge. We expect that the Hcy-Mo probe will show excellent biocompatibility and fluorescence response performances.

### Time response

**Fig. 1:**
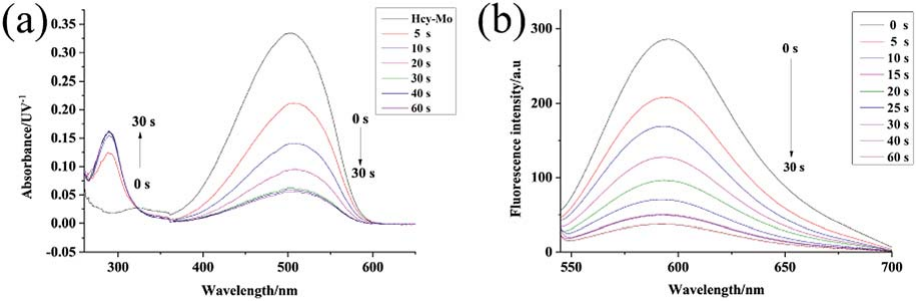
Absorption (a) and fluorescence spectral (b) changes with the addition of the Hcy-Mo probe (10 μM) in the absence and presence of sodium bisulfite (10 equiv.) in buffer solution (10 mM PBS, pH = 7.4) with *λ*_ex_ of 510 nm at 25 °C.

The absorption spectral properties of Hcy-Mo in the absence and presence of bisulfite in the buffer are shown in Fig. 1. When 10 equiv. of bisulfite was added, the maximum absorption of Hcy-Mo at 503 nm decreased remarkably and a new absorption peak at 280 nm increased gradually, with a well-defined isosbestic point at 320 nm. At the same time, the color of the solution changed from brown to colorless, which could be seen by the naked eye. Moreover, the addition of sodium bisulfite also resulted in a remarkable decline in fluorescence at 596 nm upon excitation at 510 nm.

### Sensitivity research

**Fig. 2:**
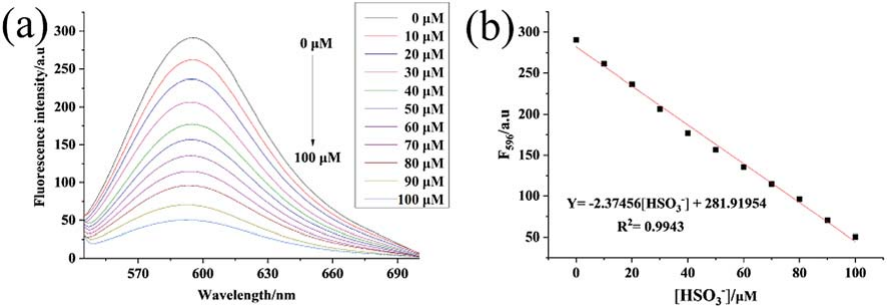
(a) Fluorescence spectra of Hcy-Mo (10 μM) upon the addition of increasing concentrations of sodium bisulfite (0, 10, 20, 30, 40, 50, 60, 70, 80, 90, and 100 μM) when excited at 510 nm at 25 °C. (b) Fluorescence intensity changes of Hcy-Mo at 596 nm as a function of sodium bisulfite concentration.

The emission behavior of the Hcy-Mo probe (10 μM) in the presence of bisulfite (0, 10, 20, 30, 40, 50, 60, 70, 80, 90, and 100 μM) was investigated, as shown in Fig. 2. The fluorescence intensity at 596 nm decreased with increasing bisulfite concentration. By plotting the fluorescence intensity of the Hcy-Mo probe at 596 nm *versus* the concentration of bisulfite, a good linear relationship (*R*^2^ = 0.994) was obtained with concentrations ranging from 0 to 100 μM. The detection limit was calculated to be 80 nM using the detection limit formula LOD = 3*δ*/*k*. It is worth mentioning that Hcy-Mo exhibited a high quantum yield (*Φ*_F_ = 0.22) in aqueous medium when excited at the *λ*_max_ (510 nm) of Hcy-Mo.

### Selectivity tests

**Fig. 3:**
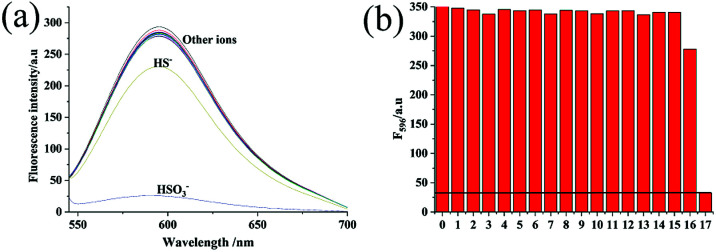
(a) Fluorescence responses and (b) fluorescence enhancements at 596 nm of Hcy-Mo (10 μM) to 10 equiv. of sodium bisulfite and 20 equiv. of interfering analytes (0: Hcy-Mo, 1: F^−^, 2: Cl^−^, 3: Br^−^, 4: I^−^, 5: HCO_3_^−^, 6: CO_3_^2−^, 7: CH_3_COO^−^, 8: S_2_O_3_^2−^, 9: SO_4_^2−^, 10: NO_2_^−^, 11: N_3_^−^, 12: SCN^−^, 13: CN^−^, 14: Cys, 15: GSH, 16: HS^−^, 17: HSO_3_^−^).

Among all the factors, the selectivity of a probe is the most important one because of the complexity of biological samples. For this reason and on the basis of the above results, we further evaluated the selectivity of Hcy-Mo probe toward bisulfite over other relevant ions and amino acids including NaF, NaCl, NaBr, NaI, NaHCO_3_, Na_2_CO_3_, CH_3_COONa, Na_2_S_2_O_3_, Na_2_SO_4_, NaNO_2_, NaN_3_, NaSCN, cysteine, glutathione, tetrabutyl cyanide ammonium, and NaHS. As shown in Fig. 3, compared with many ions, only HS^−^ exhibited a little decrease in the fluorescence intensity of Hcy-Mo. Furthermore, sodium bisulfite (100 μM) caused a reduction in the Hcy-Mo (10 μM) peak intensity at 596 nm, which is a 5-fold decrease compared with sodium hydrosulfide. Thus, the Hcy-Mo probe showed good selectivity towards bisulfite.

### pH stability

In order to explore *in vivo* imaging potential, Hcy-Mo was placed under different pH conditions and the changes in fluorescence intensity at 596 nm with the absence and presence of bisulfite were measured. As shown in Fig. S1,† pH did not exert much influence on the fluorescence intensity of Hcy-Mo over the range of 7–9.

### Mechanism verification

**Fig. 4:**
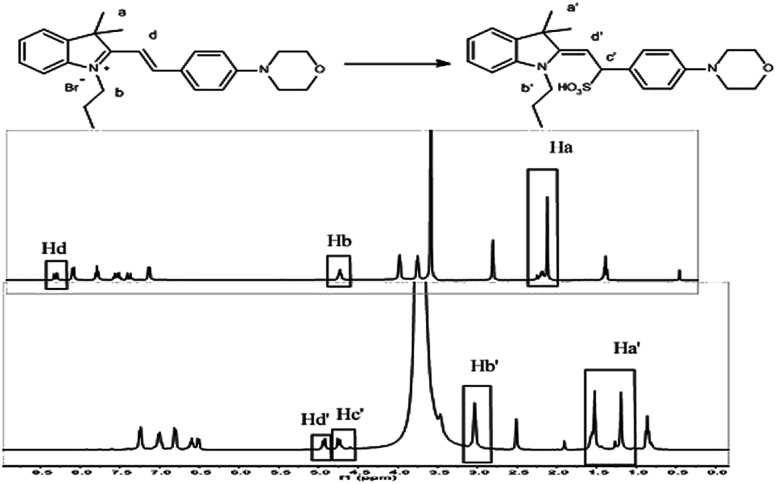
^1^H NMR titration of Hcy-Mo probe with sodium bisulfite.

The detection mechanism of the Hcy-Mo probe toward bisulfite was speculated using data collected from ^1^H NMR spectroscopy. As shown in Fig. 4, the quaternary ammonium changed to a tertiary amine due to a 1,4-addition reaction between the bisulfite and the quaternary ammonium salt. Thus, the chemical shift value of H_b_ changed from 4.5 ppm to 3.0 ppm. Meanwhile, the chemical shift value of H_d_ moved from 8.2 ppm to 5.0 ppm because the conjugated bridge was broken. Moreover, due to the strong electron-withdrawing effect of bisulfite, a new signal peak at 4.7 ppm (H_c′_) appeared. Thereby, the flow of electrons in the entire molecule was destroyed and the molecular fluorescence was quenched.

To further confirm the detection mechanism, Gaussian 09W software was used for theoretical calculations. As shown in Fig. S3 and S4,† the optimal configuration of Hcy-Mo and Hcy-Mo + HSO_3_^−^ was obtained using density functional theory with 6-31G as the basis set. Significant optimal configuration differences appeared for Hcy-Mo after the addition of HSO_3_^−^, including the breakage of the conjugated bridge, changes to bond angles, and removal of co-planarity between the electron donor and acceptor. Moreover, the results of LUMO and HOMO were visualized, as shown in Fig. S2.† The π electrons in the HOMO of the probe were mainly located on the benzene ring and N atom of morpholine, while the electrons in the LUMO were situated on indolizidine. Thus, ICT occurred between the donor and acceptor units resulting in strong fluorescence. However, after treatment with bisulfite, the π electrons in the HOMO of Hcy-Mo + HSO_3_^−^ were located on bisulfite, while the electrons in the LUMO were positioned on the benzene ring. Therefore, the strong fluorescence emission disappeared because of the suppression of electron transfer.

### *In vitro* paper test

**Fig. 5:**
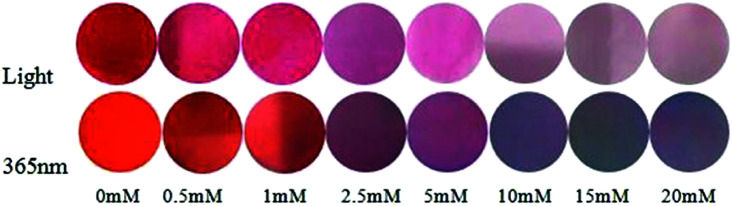
Hcy-Mo (1 mM) with different concentrations of sodium bisulfite (0, 0.5, 1, 2.5, 5, 10, 15, and 20 mM) in sunlight and 365 nm fluorescent light.

Encouraged by the high sensitivity and selectivity of the Hcy-Mo probe, we evaluated its potential for the on-site visual detection of an aqueous solution using a colorimetric tube and probe-coated test papers. As shown in Fig. S3,† the color changed from brown to colorless with the addition of bisulfite but no color change was observed with the addition of other ions. Moreover, as shown in Fig. 5, the color of the round test strips changed from brown to white with increasing concentration of bisulfite. Meanwhile, the color changed from purple to blue under a UV lamp (365 nm). Thus, the results showed that Hcy-Mo possessed detection potential for bisulfite in aqueous solution.

### Cytotoxicity and cell imaging

**Fig. 6:**
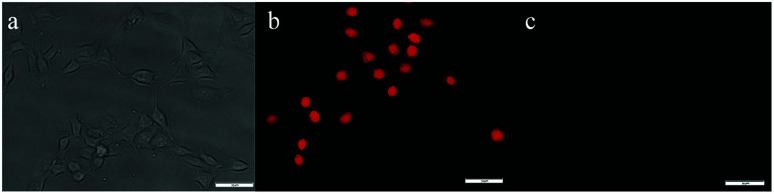
Cell imaging of MDA-MB-231 cells treated with Hcy-Mo (10 μM) in bright filed (a), absence (b) and presence (c) of sodium bisulfite (100 μM) in the red channel (*λ*_ex_ = 510 nm, *λ*_em_ = 580–660 nm) scale bar: 50 μm.

As mentioned above, to further explore the cytotoxicity and *in vivo* fluorescence imaging of the Hcy-Mo probe, cell imaging experiments were carried out. As shown in Fig. 6, strong red fluorescence was observed when the cells were incubated with a medium containing Hcy-Mo (10 μM) for 30 min. The red fluorescence disappeared after treatment with sodium bisulfite (100 μM) for 5 min. The results showed that Hcy-Mo has potential for *in vivo* experiments.

As shown in the MTT assay in Fig. S4,† cell viability was reduced to less than 10% after 24 h of incubation at a concentration of 10 μM, which indicated that Hcy-Mo had low cytotoxicity towards MDA-MB-231 cells. Hence, Hcy-Mo shows potential for biological imaging.

### *In vivo* imaging experiments

**Fig. 7:**
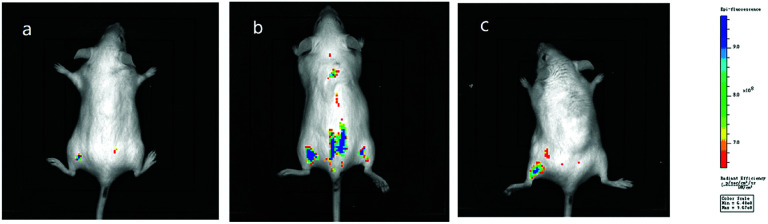
(a) *In vivo* images of a mouse without the Hcy-Mo probe; (b) *in vivo* images of the mouse with the Hcy-Mo probe (50 μM, 20 μL) in the absence of bisulfite; (c) *in vivo* images of the mouse with Hcy-Mo after injection of NaHSO_3_ (500 μM, 20 μL).

Encouraged by the promising profiles described above, *in vivo* imaging was carried out. Kunming mice, a typical experimental model, were divided into three groups as experimental subjects to explore the *in vivo* imaging effect of Hcy-Mo. The blank, control, and experimental group were injected with PBS, Hcy-Mo (50 μM), and Hcy-Mo (50 μM) + HSO_3_^−^ (500 μM), respectively. Tail vein injection was adopted as the injection method. As shown in Fig. 7, compared to the blank group, the control group showed strong fluorescence emission. At the same time, the fluorescence emission of the experimental group disappeared after treatment with bisulfite. In conclusion, the above data indicates that Hcy-Mo can be employed for the efficient detection of bisulfite in living mice.

## Conclusions

In conclusion, based on a hemi-cyanine scaffold and morpholine group, a bisulfite fluorescent probe (Hcy-Mo) was designed and synthesized. Hcy-Mo shows promising profiles including a fast response (within 30 s), good water solubility (in pure PBS), pH stability (7–9), and high sensitivity (LOD = 80 nM) and selectivity. In addition, low cytotoxicity and biological imaging potential are exhibited in MDA-MB-231 cells. Moreover, the detection of bisulfite in living mice was performed. The results of the experiments indicated that Hcy-Mo possessed live imaging potential, which provides a valuable tool for tracing the distribution and metabolism of bisulfite in biological systems.

## Conflicts of interest

There are no conflicts to declare.

## Supplementary Material

RA-010-D0RA03329D-s001
