# Supplementary material for: An effective biocompatible fluorescent probe for bisulfite detection in aqueous solution, living cells, and mice
Source: RSC Adv. 2020 Jul 3;10(42):25352–7. doi: 10.1039/d0ra03329d (PMC9055347; doi:10.1039/d0ra03329d)
Supplement: RA-010-D0RA03329D-s001 [file RA-010-D0RA03329D-s001.pdf]

Electronic Supplementary Material (ESI) for RSC Advances.
This journal is © The Royal Society of Chemistry 2020

Supporting Information

An effective biocompatible fluorescent probe for bisulfite detection in aqueous solution, living cells and mice

Ruqiao Zhou[a], Guiling Cui[b], Yuefu Hu[b], Qingrong Qi[b], Wencai Huang[c], Li Yang[a*]

[a] State Key Laboratory of Biotherapy and Cancer Center, West China Hospital, Sichuan University, Chengdu, Sichuan 610041, P.R. China
[b] West China School of Pharmacy, Sichuan University, Chengdu, 610041
[c] School of Chemical Engineering, Sichuan University, Chengdu, 610065

[*]Corresponding author: Li Yang

Email address: yangli@scu.edu.cn

Contents

1. **The synthesis route of probe Hcy-Mo** 2
2. **Additional of Hcy-Mo** 3
3. **Optimal configuration** 4
4. **NMR spectra and HR-ESI-MS spectrum of compound 1, 2, 3, Hcy-Mo** 5

1. The synthesis route of probe Hcy-Mo

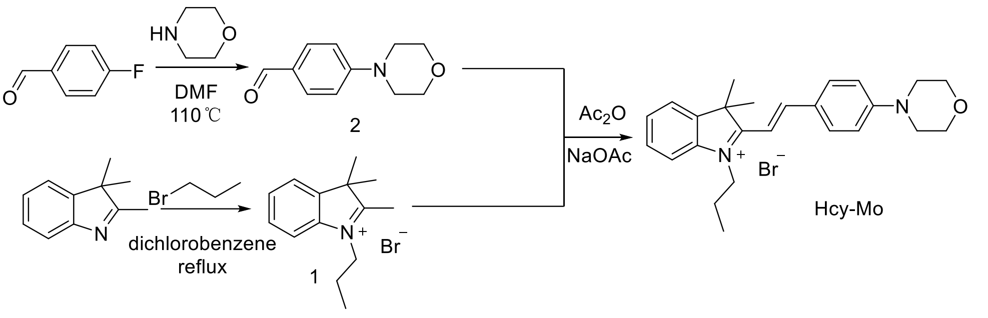

Scheme. S1 Synthesis of probe Hcy-Mo

Synthesis of Compound 1

Added 10.28 g 2,3,3-Trimethyl indolenine (64.18 mol, 1 equive) and 11.83 g n-propyl bromide (96.27 mmol, 1.5 equive) to a 100mL single-mouth reaction flask. After that, add 20 mL of o-dichlorobenzene, and then stirred and reflux at 110 ° C for 12 h under nitrogen protection. After the reaction is finished, cooled to room temperature, add 100 mL of isopropyl ether and stir, there are sticky substance adhere at the bottom of the bottle, poured out the solvent, added little acetone and stirred for 15 min, a large amount of purple solid was precipitated. After suction filtration, the cake layer was washed with a small amount of acetone for 3~4 times, and then dried under vacuum to obtain a white solid product of 6.88 g. Yield was 37.8%.

^{1}HNMR (400 MHz, DMSO) δ 8.04 – 7.98 (m, 1H), 7.88 – 7.82 (m, 1H), 7.67 – 7.58 (m, 2H), 4.45 (t, J = 7.5 Hz, 2H), 2.86 (s, 3H), 1.88 (dd, J = 15.0, 7.5 Hz, 2H), 1.55 (s, 6H), 1.00 (t, J = 7.4 Hz, 3H).

Synthesis of Compound 2

Take p-Fluorobenzaldehyde 2.48 g (20 mmol, 1 equive), morpholine 1.91 g (22 mmol, 1.1 equive), anhydrous potassium carbonate 5.52 g (40 mmol, 2 equive) into a 100 ml single-mouth reaction bottle. After adding 30 ml of anhydrous DMF, the mixture was refluxed at 140 ° C for 2~4 h, the reaction was monitored by TLC. After the reaction was completed, the heating was stopped, the mixture was poured into ice water, stirred at room temperature for 20 min, and a large amount of solid was precipitated and filtered. The crude product was purified by column chromatography (dichloromethane) to afford 2.6 g of pale yellow solid. yield was 68.1%.

^{1}HNMR (400 MHz, $CDCl_3$) δ 9.80 (s, 1H), 7.80 – 7.75 (m, 2H), 6.94 – 6.90 (m, 2H), 3.88 – 3.83 (m, 4H), 3.37 – 3.33 (m, 4H).^{13}CNMR (100 MHz, $CDCl_3$) δ 190.45, 155.15, 131.80, 127.70, 113.48, 66.50, 47.32.

Synthesis of Hcy-Mo

4-morpholine benzaldehyde 306mg (1.6 mmol, 1.1 equive), 2,2,3-trimethylsulfonium bromide 421mg (1.5 mmol, 1equive), anhydrous sodium acetate 120 mg (1.5 mmol, 1 equive) was added to a 25 ml single-mouth reaction flask. The mixture was refluxed at 85 ° C for 1~2 h under nitrogen atmosphere after added 6 ml of anhydrous acetic anhydride. The reaction was monitored by TLC. After the reaction was completed, stopped heating and cooled to room temperature, and then poured into a saturated brine. Extract with dichloromethane (3×30 ml), combined the organic phases and concentrated. then added 30 ml of isopropyl ether and filtered, the filter cake was washed 3~4 times with isopropyl ether, then dried in vacuo to give a purple solid product 320 mg, yield 46.9%.

^{1}HNMR (400 MHz, DMSO-d_6) δ 8.37 (d, J = 15.8 Hz, 1H), 8.13 (d, J = 8.7 Hz, 2H), 7.81 (t, J = 7.8 Hz, 2H), 7.57 (t, J = 7.2 Hz, 1H), 7.52 (t, J = 7.3 Hz, 1H), 7.39 (d, J = 15.8 Hz, 1H), 7.11 (d, J = 8.7 Hz, 2H), 4.55 (t, J = 7.2 Hz, 2H), 3.75 (t, J = 4.7 Hz, 4H), 3.51 (t, J = 4.8 Hz, 4H), 1.85 (q, J = 7.3 Hz, 2H), 1.77 (s, 6H), 1.00 (q,

J = 7.5 Hz, 3H).^{13}CNMR (100 MHz, DMSO-d_6) δ 180.85, 155.10, 154.88, 143.51, 141.51, 134.45, 129.35, 128.51, 124.43, 123.37, 114.65, 113.89, 106.94, 66.27, 51.78, 47.10, 46.87, 26.89, 21.92, 11.27. ESI-MS m/z calcd for Chemical Formula: $C_{25}H_{31}N_2O[M]^+$: 375.2191; found: 375.2184.

2. Additional of Hcy-Mo

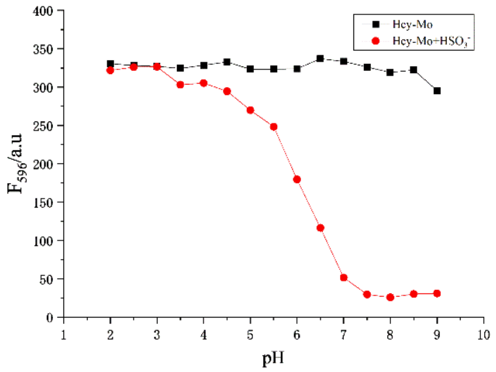

Fig. S1. luorescence intensity changes of **Hcy-Mo** at 596 nm upon the addition of sodium bisulfite(10 equiv) in different pH

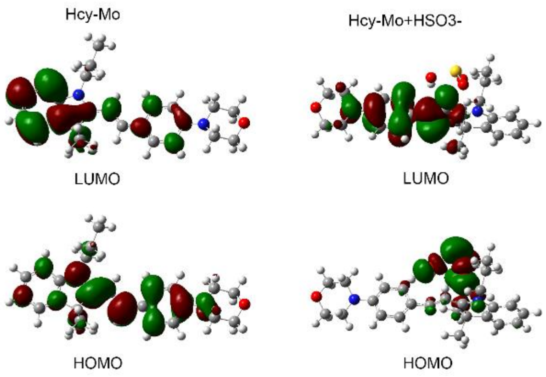

Fig. S2. HOMO and LUMO simulated by Gaussian 09W (red, gray, yellow, blue and white globe respectively represent O, C, S, N, H atoms)

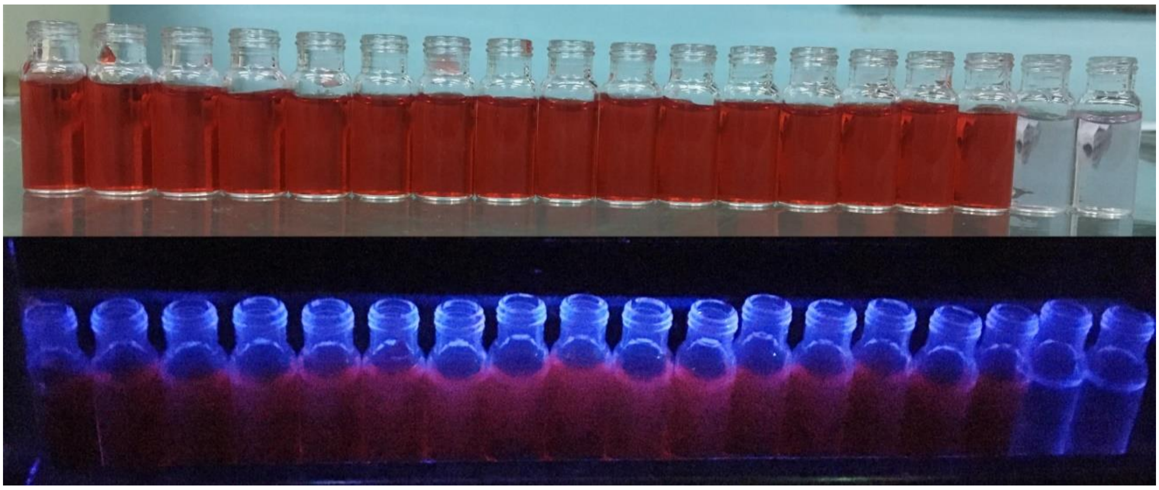

Fig. S3. **Hcy-Mo** with different ions in the daylight and 365 nm fluorescent light

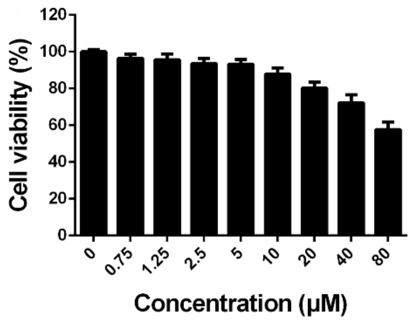

Fig. S4 MDA-MB-231 cell viability to different concentration of **Hcy-Mo**

3. Optimal configuration

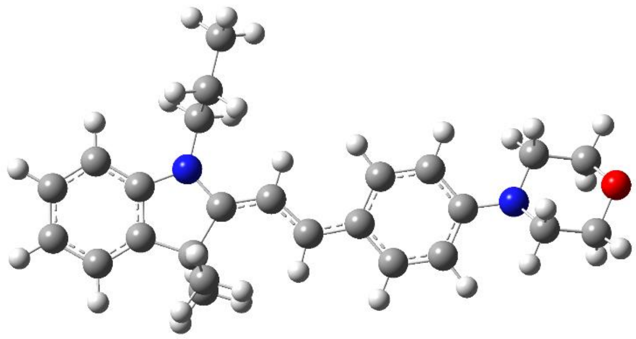

Fig. S5. Optimal configuration of Hcy-Mo calculated by Gaussian 09W

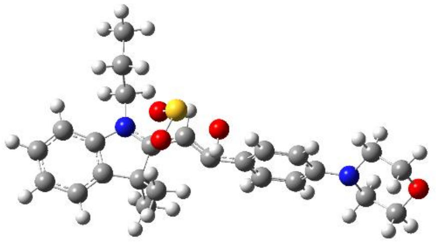

Fig. S6. Optimal configuration of Hcy-Mo+HSO_3^- calculated by Gaussian 09W

4. NMR spectra and HR-ESI-MS spectrum of compound 1, 2, 3, Hcy-Mo

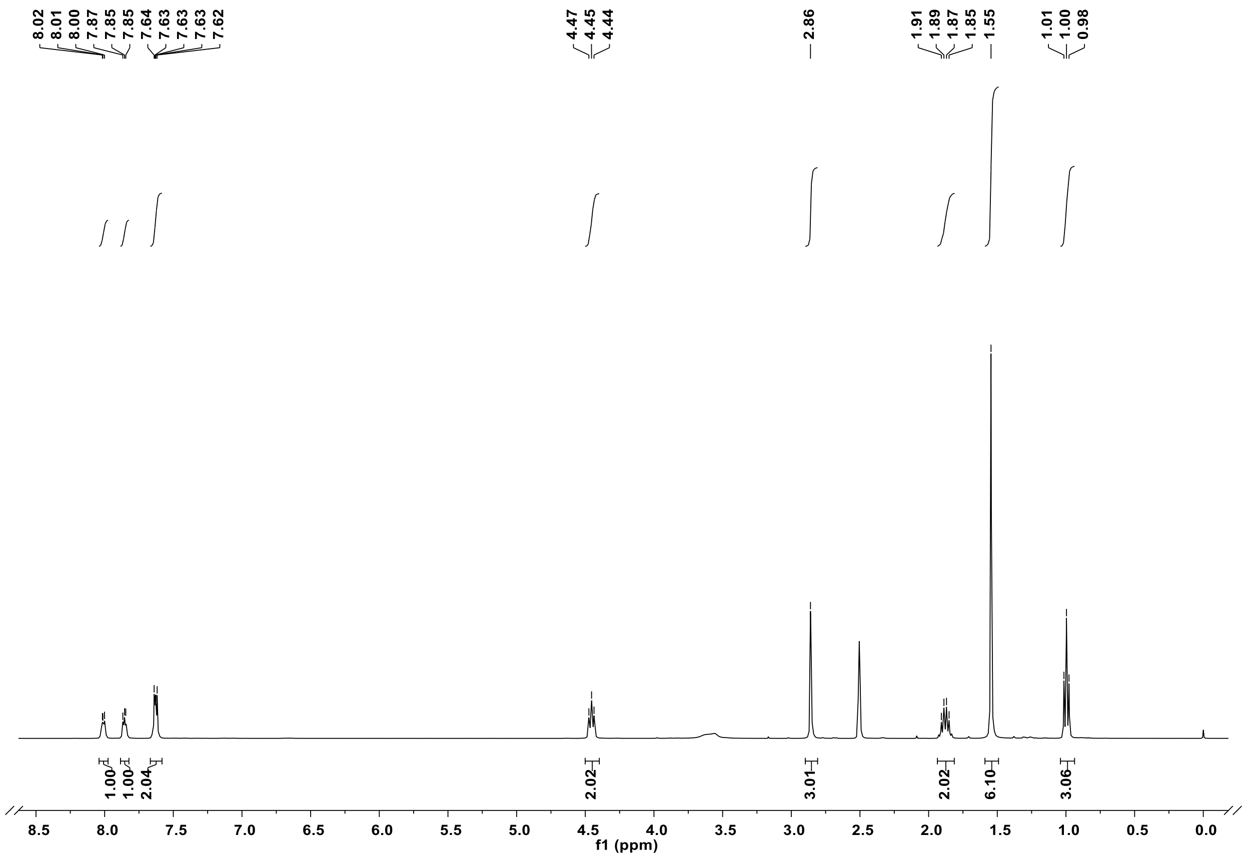

Fig. S7. ^{1}HNMR spectrum(400 MHz) of compound 1 in DMSO

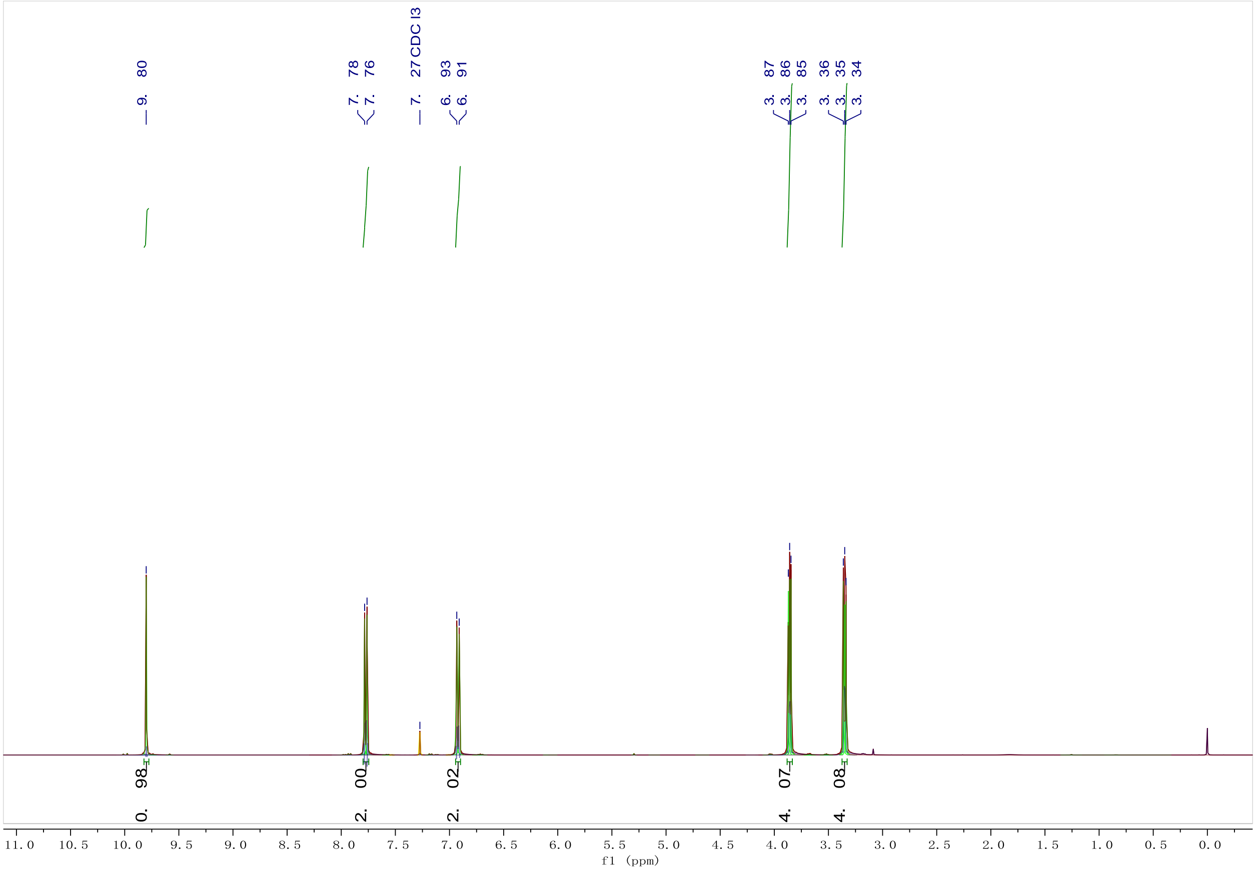

Fig. S8. ^{1}HNMR spectrum(400 MHz) of compound 2 in $CDCl_3$

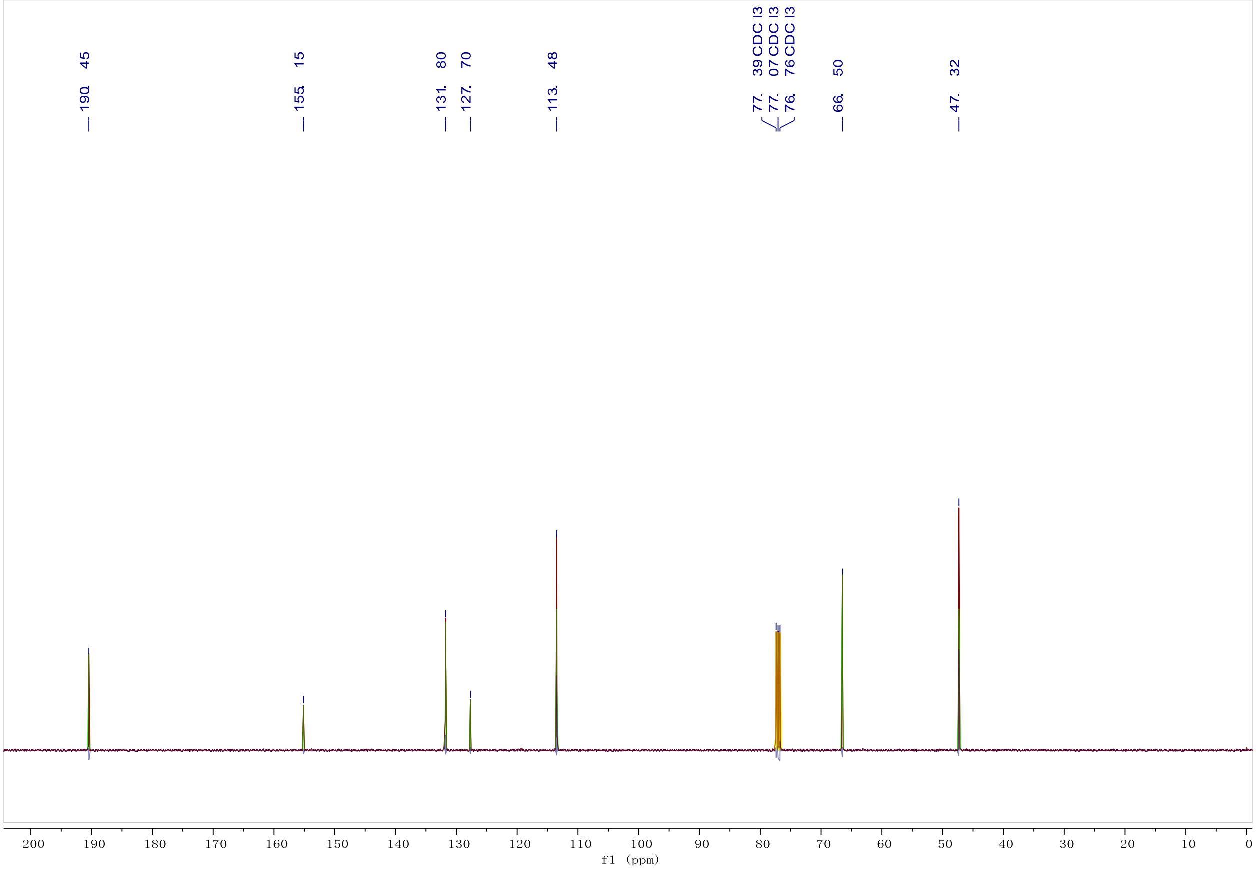

Fig. S9. ^{13}CNMR spectrum (100 MHz) of compound 2 in $CDCl_3$

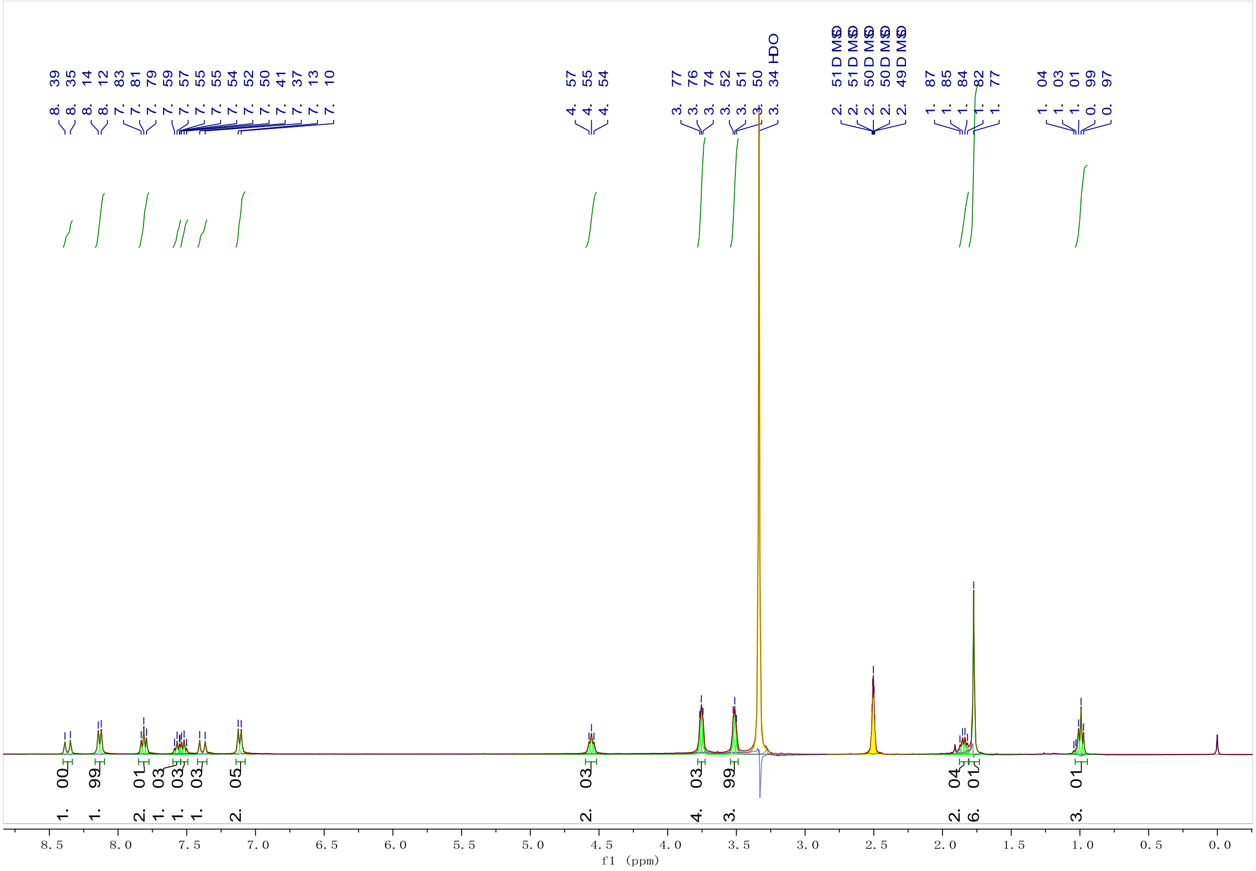

Fig. S10. ^{1}HNMR spectrum (400 MHz) of Hcy-Mo in DMSO

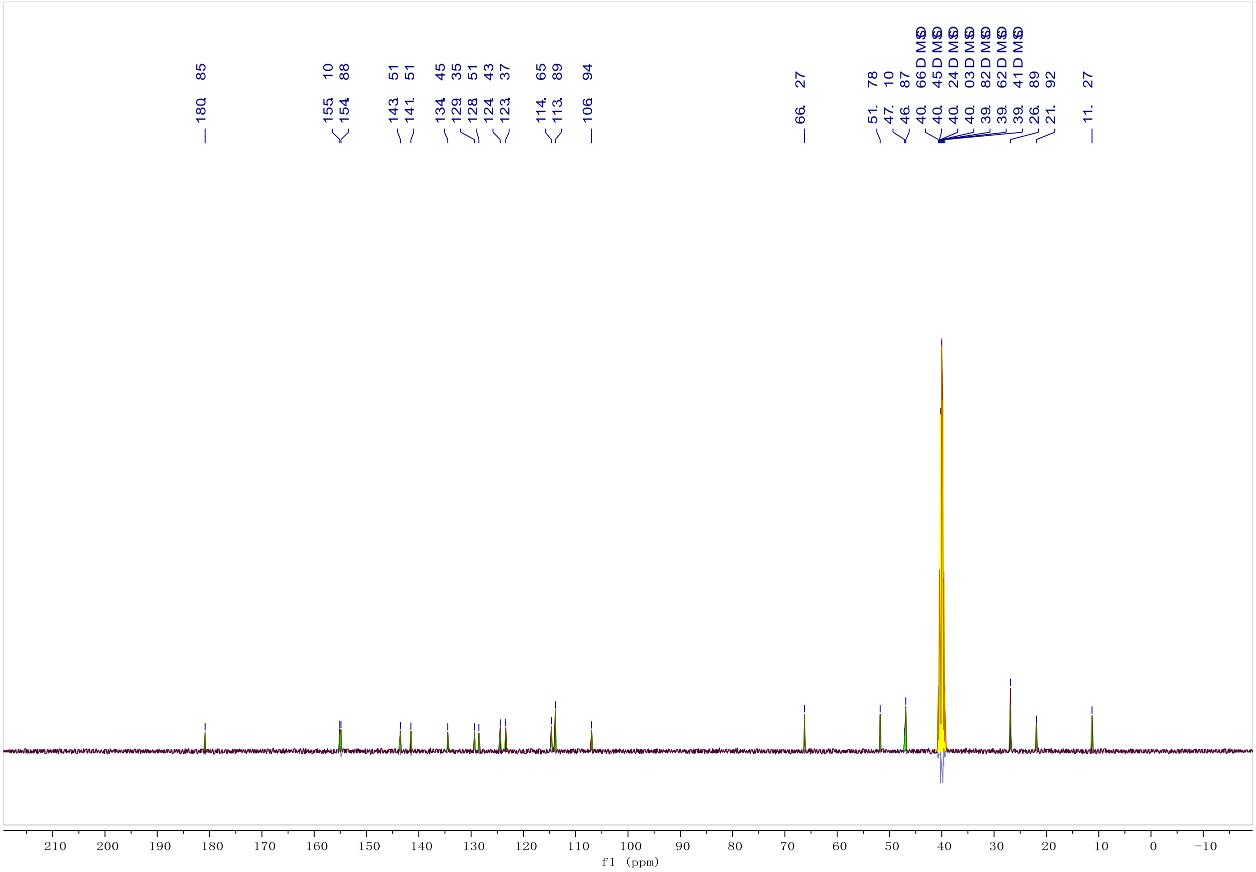

Fig. S11. ^{13}CNMR spectrum (100 MHz) of Hcy-Mo in DMSO

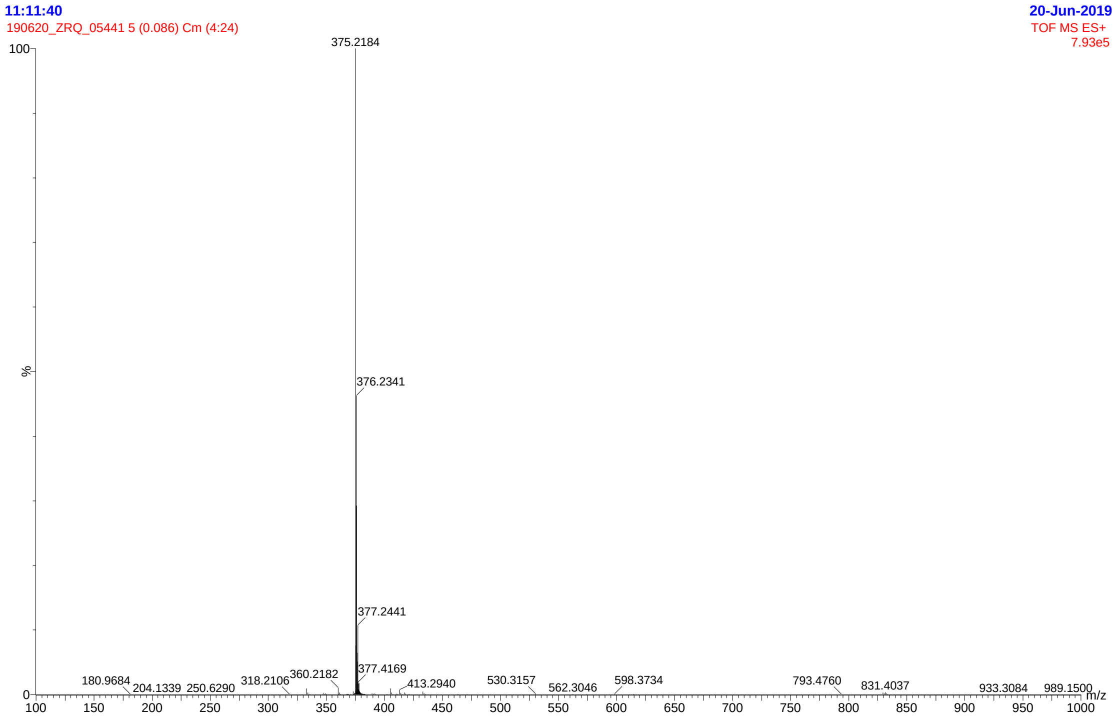

Fig. S12. HR-ESI-MS spectrum of Hcy-Mo
